# Postoperative circadian patterns in wearable sensor measured heart rate: a prospective observational study

**DOI:** 10.1007/s10877-023-01089-z

**Published:** 2023-10-21

**Authors:** Eveline H.J. Mestrom, Jonna A. van der Stam, Simon W. Nienhuijs, Ignace H.J.T. de Hingh, Arjen-Kars Boer, Natal A.W. van Riel, Volkher Scharnhorst, R. Arthur Bouwman

**Affiliations:** 1 Department of Electrical Engineering, Eindhoven University of Technology, Eindhoven, The Netherlands; 2 Department of Anesthesiology, Intensive Care & Pain Medicine, Catharina Hospital, Eindhoven, The Netherlands; 3 Department of Biomedical Engineering, Eindhoven University of Technology, Eindhoven, The Netherlands; 4 Clinical laboratory, Catharina Hospital, Eindhoven, The Netherlands; 5 Expert Center Clinical Chemistry Eindhoven, Eindhoven, The Netherlands; 6 Department of Surgery, Catharina Hospital, Eindhoven, The Netherlands; 7 GROW – School for Oncology and Developmental Biology, Maastricht University, Maastricht, The Netherlands; 8 Department of Vascular Medicine, Amsterdam University Medical Centers, Amsterdam, The Netherlands

**Keywords:** Circadian patterns, Diurnal rhythm, Continuous monitoring, Deterioration, Wearable sensor, Postoperative patients, Early warning score

## Abstract

**Purpose:**

This study aimed to describe the 24-hour cycle of wearable sensor-obtained heart rate in patients with deterioration-free recovery and to compare it with patients experiencing postoperative deterioration.

**Methods:**

A prospective observational trial was performed in patients following bariatric or major abdominal cancer surgery. A wireless accelerometer patch (Healthdot) continuously measured postoperative heart rate, both in the hospital and after discharge, for a period of 14 days. The circadian pattern, or diurnal rhythm, in the wearable sensor-obtained heart rate was described using peak, nadir and peak-nadir excursions.

**Results:**

The study population consisted of 137 bariatric and 100 major abdominal cancer surgery patients. In the latter group, 39 experienced postoperative deterioration. Both surgery types showed disrupted diurnal rhythm on the first postoperative days. Thereafter, the bariatric group had significantly lower peak heart rates (days 4, 7–12, 14), lower nadir heart rates (days 3–14) and larger peak-nadir excursions (days 2, 4–14). In cancer surgery patients, significantly higher nadir (days 2–5) and peak heart rates (days 2–3) were observed prior to deterioration.

**Conclusions:**

The postoperative diurnal rhythm of heart rate is disturbed by different types of surgery. Both groups showed recovery of diurnal rhythm but in patients following cancer surgery, both peak and nadir heart rates were higher than in the bariatric surgery group. Especially nadir heart rate was identified as a potential prognostic marker for deterioration after cancer surgery.

## Introduction

Circadian patterns or diurnal rhythmicity are well recognized in many physiological systems [1–3]. While physicians are aware of these daily patterns, they are often not incorporated into the interpretation of patient values. For example, the heart rate in postoperative patients is assessed during manual spot checks that are typically performed once every eight hours during ward rounds. From these vital parameters, early warning scores are calculated [4]. These spot checks are usually performed when a patient is either awake or awakened even though this waking state might influence heart rate. Additionally, early warning scores typically use a fixed-point scoring table to detect potential deterioration that does not incorporate the influence of diurnal rhythm.

Previous literature describes the use of biomarkers such as serum melatonin and cortisol levels, polysomnography and temperature to describe diurnal rhythm [5–8]. However, repeated collection of biomarkers or continuous monitoring with wired sensors is challenging to implement in clinical practice, especially in low-care environments such as the general ward or at home. Novel wearable sensors provide an attractive source of data for the assessment of diurnal rhythm, as they are able to continuously and unobtrusively collect vital parameters.

More insight into the postoperative course of vital parameters would be especially interesting, as the literature describes disruption of the diurnal rhythm following surgery and anaesthesia [9–12], and the disturbance of the diurnal timing system might negatively affect an individual’s ability to recover after surgery [12]. This hypothesis is supported by a study by Davidson et al., who were the first to study diurnal rhythms in continuous measurements of vital parameters in the ICU, where they described potential prognostic value for patient recovery [13]. New innovations, such as continuous monitoring using wearables, have been increasingly investigated and recent studies have shown the potential for deterioration detection in perioperative care [14–17]. While wearables allow for continuous monitoring of heart rate regardless of the state of consciousness and the patient’s location, the diurnal rhythm of heart rate during normal recovery and its potential impairment in complicated postoperative recovery remain unknown.

As insight into postoperative vital parameters outside the ICU is limited, the aim of this study is twofold. First, we describe the postoperative diurnal rhythm of heart rate in patients with deterioration-free recovery. Second, we compared the postoperative diurnal rhythm in heart rate of patients with deterioration-free recovery to patients experiencing postoperative deterioration.

## Methods

### Study population

The present work describes the results of the TRICA study NCT03923127, a single-center study on wearable sensors in postoperative patients in a tertiary hospital (Catharina Hospital, Eindhoven, The Netherlands). The trial was approved by the medical ethical committee (W19.001).

The study population consisted of 350 patients who were scheduled for either major abdominal cancer surgery or bariatric surgery between April 2019 and August 2020. Patients were not included if they met any of the following exclusion criteria: pregnant or breastfeeding, younger than 18 years, allergy to tissue adhesives, antibiotic-resistant skin infection, active implantable device or any skin condition at the area of application of the devices. Additionally, patients signed informed consent forms prior to the start of the research procedures.

### Wearable sensor

The study population wore the investigational device Healthdot (Philips Electronic Netherlands B.V.), a wearable patch of 5 × 3 cm that weighs 13.6 g and is applied to the patient’s left lower rib on the midclavicular line. The accelerometer-based device can measure the vital parameters heart rate and respiratory rate for up to two weeks. The built-in wireless long-range communication technology is able to send 5-minute averages of the vital parameters to a cloud server. The clinical accuracy of the device in postoperative bariatric and abdominal cancer surgery patients has previously been described [18, 19]. In the present work, only the heart rate was considered, which was stored in the internal memory of the patch at time intervals of 8 s. After extraction from the internal memory the data were resampled to a 1 s interval using linear interpolation. All vital parameters are reported together with a date-time stamp expressed in GMT and a quality index (range: 0–100).

The wearable sensor was applied immediately after the surgical procedure while the patient was either in the postanaesthesia care unit or intensive care unit. The device was then worn for up to two weeks, both in the hospital and at home. During the data collection, both the regular care team and research team were blinded to the data collected by the wearable patch.

### Preprocessing

Preprocessing of the wearable sensor data included exclusion of low-quality measurements (quality index = 0). Thereafter, the HR measurements were downsampled to 5-minute averaged data to match the sampling frequency of the cloud data storage transmissions. Preoperative HR was extracted from the electronic medical records, and the closest heart rate recording at least 7 days before the day of surgery was used for analysis.

### Data analysis

Only patients with at least the first 7 consecutive days of wearable sensor data were included in the analysis. Characteristics of the population were expressed as the mean and standard deviation (SD) in the case of normally distributed data or the median and interquartile range (IQR) in the case of nonnormally distributed data. Binominal variables are expressed as numbers and percentages. Statistical comparisons were performed using an unpaired t test for normally distributed data, a Mann-Whitney test for nonnormally distributed data and a Chi-squared test for binominal data. A p-value < 0.05 was considered statistically significant.

The diurnal rhythm in the course of the postoperative via wearable patch-collected HR was expressed using peak-nadir excursions [20]. To avoid the major influence of outliers or measurement errors, the peak was defined as the 95th percentile of the data, and the nadir was defined as the 5th percentile. Consequently, the peak-nadir excursions describe the difference between the 95th and 5th percentiles.

First, the normal postoperative course of HR and peak, nadir and peak-nadir excursions in bariatric and major abdominal cancer surgery patients with deterioration-free recovery was studied. In order to asses which patients experienced a postoperative complication, all complications in the postoperative period were logged in the electronic Case Report Form with their associated Clavien-Dindo (CD) score [21]. In the analyses, all patients without a deterioration defined as a complication with a CD of 2 or higher were evaluated. Second, the postoperative course of HR in major abdominal cancer surgery patients who experienced a postoperative deterioration (CD ≥ 2) was compared to that in major abdominal cancer surgery patients without deterioration. To assess the potential predictive value of the HR and diurnal rhythm features, only data until deterioration were used in the visualization of the postoperative HR, and only data until the day of deterioration were used in the analysis of the diurnal rhythm features. Therefore, if a patient experienced multiple deteriorations, only the first was considered. Due to the use of this censoring at the time of deterioration, the number of patients decreased over time. To ensure that a significant proportion of the data remained available for analysis, only data from the first five postoperative days were considered.

For both comparisons, the postoperative HR for the two groups is visualized as the median (line) and IQR (shaded area). Additionally, peak, nadir and peak-nadir excursions for the two groups are visualized as median (dots) and IQR (shaded area). Statistical comparisons of the peak, nadir and peak nadir-excursions were performed for each of the postoperative days using a Mann-Whitney test with bonferroni correction for the comparison of the three features at the same timepoint. All data analysis was performed using R (V4.0.5) and R Studio (V2022.7.0.548) [22, 23].

## Results

### Population

**Fig. 1:**
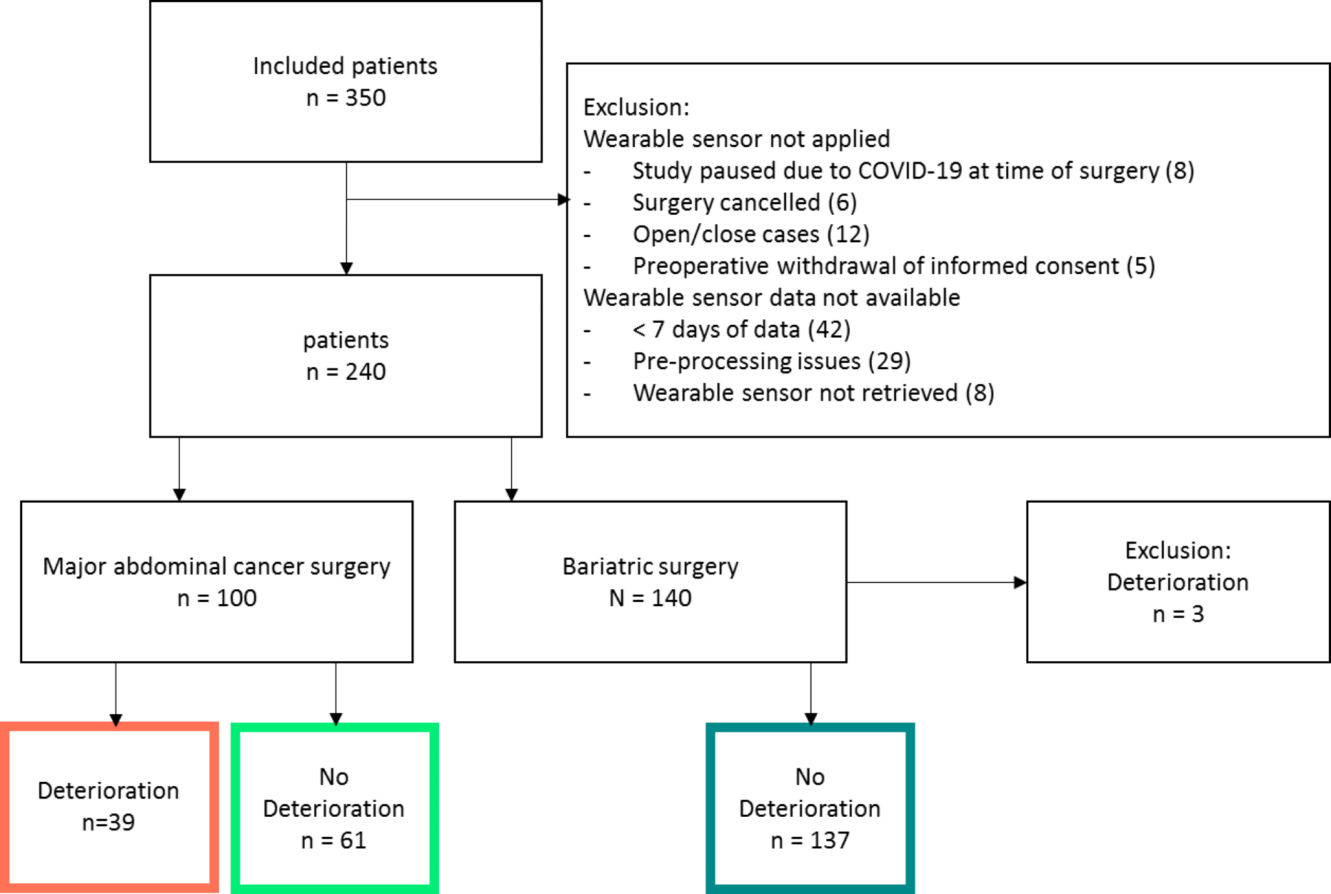
Flowchart of the study population

A total of 350 patients were enrolled in the TRICA study. The exclusion of 110 patients, as detailed in the flowchart in Fig. 1, resulted in a population of 237 patients (Fig. 1). In total, 137 (58%) of these patients underwent bariatric surgery, either a gastric sleeve (n = 58), a gastric bypass (n = 79) or a conversion from a gastric sleeve into a bypass (n = 3). The remaining 100 patients (42%) underwent major abdominal cancer surgery: either oesophageal/gastric resection (n = 24), cytoreductive surgery and HIPEC (n = 22), pancreatic surgery (n = 19), rectal surgery with (n = 19) and without (n = 7) IORT, or debulking for ovarian cancer (n = 9).

**Table 1:** Characteristics and outcomes of the patient population. The column “P surgery type” describes the outcomes of statistical tests comparing the “Cancer surgery no deterioration” and “Bariatric surgery no deterioration” columns. The column “P deterioration” describes the outcomes of the statistical tests comparing the “Cancer surgery no deterioration” and “Cancer surgery deterioration” columns

	Cancer surgery No deterioration	Bariatric surgery No deterioration	P surgery type	Cancer surgery deterioration	P deterioration
N	61	137		39	
Male sex	32 (52%)	46 (34%)	**0.01**	25 (64%)	0.25
Age in years	63 (56–69)	48 (38–55)	**< 0.01**	65 (59–71)	0.16
BMI, kg/m2	26 (4)	40 (4)	**< 0.01**	27 (5)	0.21
Preoperative heart rate in bpm	75 (12)	79 (13)	0.07	78 (13)	0.21
Preoperative use of anti-arrhythmic drugs	10 (16%)	24 (18%)	0.85	7 (18%)	0.84
ASA score					
II	49 (80%)	33 (24%)	**< 0.01**	27 (69%)	0.21
III	12 (20%)	104 (76%)	**< 0.01**	11 (28%)	0.32
Duration of surgery, in min	323 (266–395)	72 (60–86)	**< 0.01**	317 (270–378)	0.77
Postoperative IC admission	47 (77%)	0 (0%)	**< 0.01**	33 (85%)	0.36
Duration of postoperative IC admission in days	1 (1–2)			1 (1–2)	0.47
Duration of hospital admission in days	8 (7–10)	1 (1–1)	**< 0.01**	12 (8–20)	**< 0.01**

### Postoperative heart rate in uncomplicated recovery

Recovery was deterioration-free in 137 out of 140 (98%) bariatric patients compared to 61 out of 100 (61%) oncological patients. Table 1 includes the characteristics of the bariatric and cancer surgery patients who did not experience deterioration. Bariatric patients were younger and had a higher body mass index (BMI), shorter surgery duration, shorter postoperative hospital admission and higher ASA score than operated cancer patients. Cancer patients were admitted to the ICU immediately after surgery in 77% of the cases, while bariatric patients were all transferred to the post anaesthesia care unit after surgery. Preoperative heart rate did not differ significantly between the bariatric and cancer surgery subgroups.

**Fig. 2:**
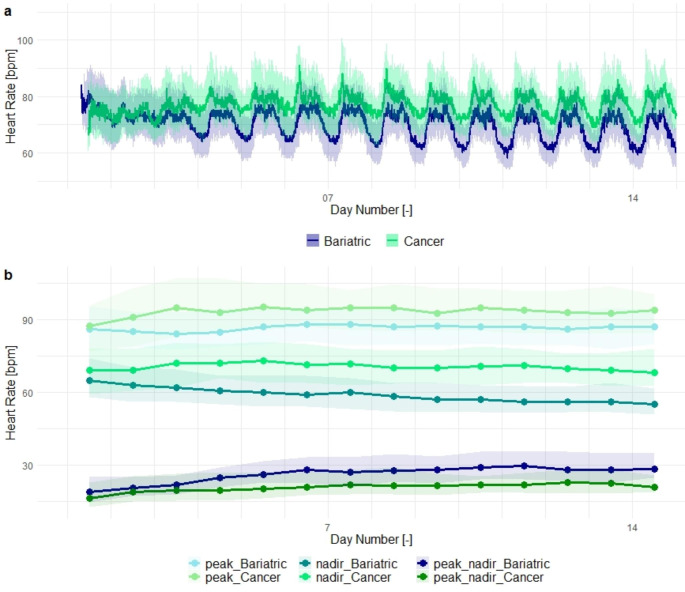
(**a**) Postoperative heart rate of the bariatric (blue) and cancer surgery (green) subgroups. The line indicates the median heart rate, and the shaded area indicates the IQR. (**b**) Postoperative peak, nadir and peak-nadir excursions of the heart rate of the bariatric (blue) and cancer surgery (green) subgroups. Dots indicate the daily median value for the respective features, and shaded areas indicate the IQR.

Figure 2a shows the postoperative heart rate of the population with both postoperative cancer surgery and bariatric surgery patients. On the first day, the data for the bariatric group is available slightly earlier than that of the cancer surgery group. This is caused by earlier application of the sensor due to the shorter duration of surgery in the first group. The median heart rate for bariatric and cancer surgery patients showed similar values on the first few postoperative days. Thereafter, the median heart rate of the bariatric population was lower than that of the cancer surgery patient group. During the first postoperative days, the median values of the subpopulations do not show a clear diurnal rhythm. A few days after surgery, both groups showed a pattern consisting of night-time heart rates lower than daytime heart rates. These differences between the daytime and night time heart rates appear larger in bariatric patients than in cancer surgery patients.

Figure 2b shows the peak, nadir and peak-nadir excursions for both bariatric and cancer surgery patients who do not experience postoperative deterioration. In agreement with Fig. 2a, bariatric surgery patients showed a lower peak heart rate (significantly lower on days 4, 7 through 12 and 14), a lower nadir heart rate (significantly lower on days 3 through 14) and a significantly larger difference between their peak and nadir (significantly larger on days 2 and 4 through 14) than cancer surgery patients.

### Postoperative heart rate in cancer surgery patients with deterioration

In 39 out of the 100 cancer surgery patients, postoperative deterioration occurred (CD 2 n = 20; CD 3 n = 9; CD 4 n = 10). The median day these patients experienced deterioration was day 6 (IQR: 4–9). Cancer surgery patients who experienced postoperative deterioration showed no significant differences in age, gender, BMI, ASA score, preoperative heart rate, surgery duration or postoperative IC admission compared to those without postoperative deterioration (Table 1).

**Fig. 3:**
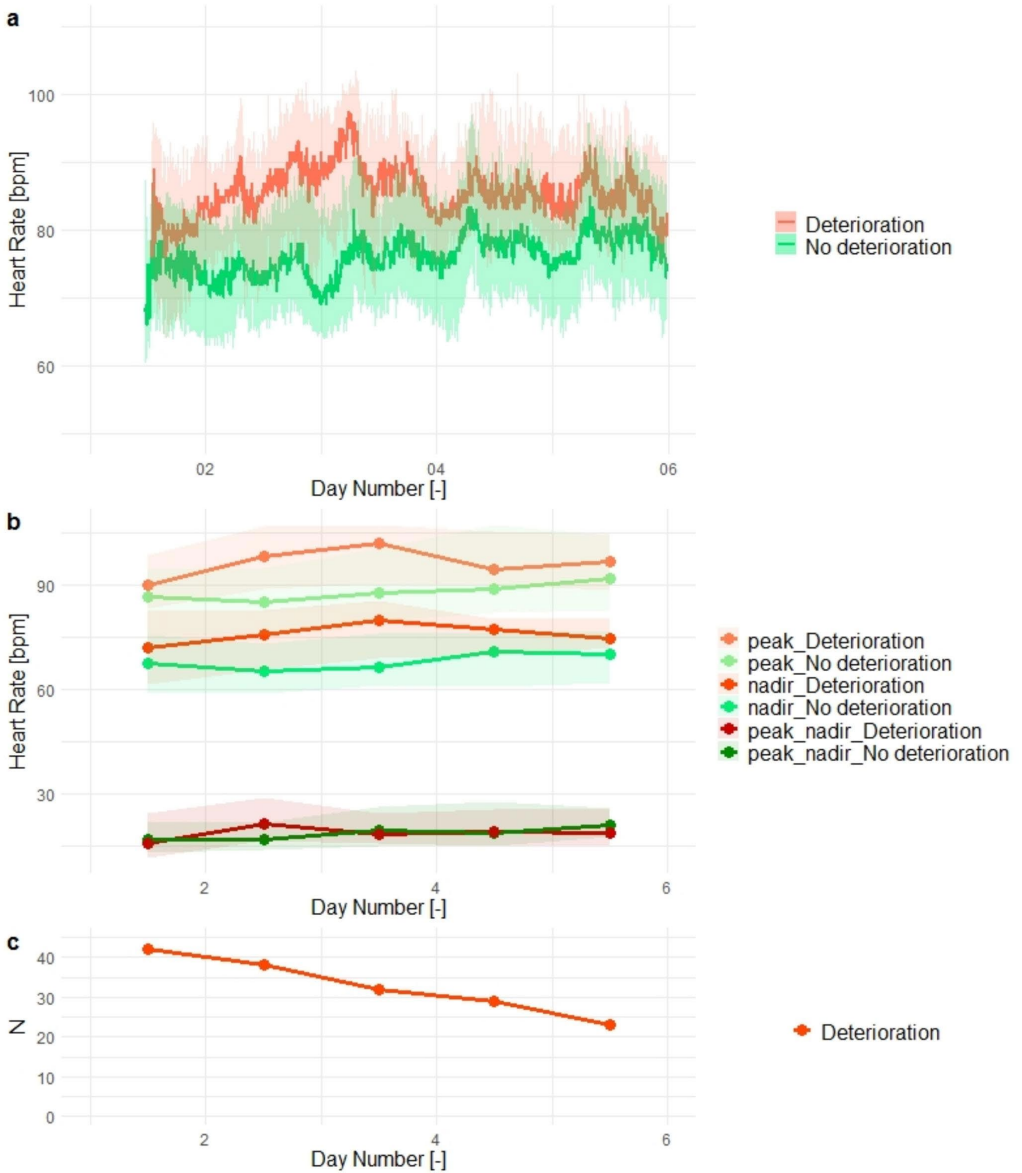
Figure 3**a**) Postoperative heart rate of major abdominal cancer surgery patients shown in subgroups of patients who experienced postoperative deterioration (red) and who had a deterioration-free recovery (green). The line indicates median heart rate, and the shaded area indicates the IQR. For patients who experienced a deterioration only data collected prior to deterioration were included. **b**) Postoperative peak, nadir and peak-nadir excursions of the heart rate of the major abdominal cancer surgery patients shown in subgroups of patients who experience postoperative deterioration (red) and who have a deterioration-free recovery (green). Dots indicate the daily median value for the respective features, and shaded areas indicate the IQR. For patients who experience a deterioration, only data collected prior to the day of their deterioration are included, leading to a reduction of the sample size for the deterioration group over time. **c**) Number of patients included in the deterioration group on each day

Figure 3a shows the postoperative heart rate for cancer surgery patients. For patients who experienced deterioration, only heart rate prior to their deterioration was included resulting in a reduction of the sample size in the deterioration group over time as illustrated in Fig. 2c. In this figure, the first few days after surgery show a higher median heart rate prior to deterioration compared to the patients with a deterioration-free recovery. Figure 3b shows the peak, nadir and peak-nadir excursions prior to deterioration and in deterioration-free recovery, again only data until the day of deterioration is included for the deterioration group. Prior to deterioration, cancer surgery patients showed a significantly higher peak heart rate on days 2 and 3 than cancer surgery patients with a deterioration-free recovery. The group that developed deterioration also had a significantly higher nadir heart rate on days 2 through 5. The difference between the peak and nadir was higher on day 2 (p = 0.03).

## Discussion

This study describes the diurnal rhythm in heart rate measured via wearable technology in postoperative patients. The first main finding in patients with deterioration-free recovery is the disruption of rhythmicity in the median heart rate during the first postoperative days for both bariatric and abdominal cancer surgery patients. The second main finding is the observation of a significantly higher nadir and peak heart rate in cancer surgery patients experiencing postoperative deterioration than in cancer surgery patients with a deterioration free recovery.

After disruption of diurnal rhythm during the first postoperative days, the mean heart rate shows a clear day and night pattern, indicating a restored diurnal rhythm. The main difference between the two types of surgery was the higher nadir and peak heart rate in cancer surgery patients, which could reflect the more pronounced invasiveness and longer duration of both the surgery and anaesthesia in these patients [11]. Bariatric patients would likely return to normal daily activity levels sooner than cancer surgery patients. This would expectedly contribute to a higher average heart rate in the bariatric cohort than would have been observed had the bariatric patients been bedridden postoperatively to the same extend as the abdominal cancer patients. Yet, bariatric patients even had a lower heart rate postoperatively. Other differences between the populations are a higher number of planned postoperative ICU admissions and longer hospital stay in cancer surgery patients. This might result in diurnal rhythm disruption due to illness, medication, artificial light or disturbed day-night schedules in the ICU [2, 13, 24, 25]. However, diurnal rhythm impairment in the bariatric group cannot be attributed to these factors since these patients were discharged on the first postoperative day. By definition, both groups suffered from different medical conditions. Compared to the bariatric group, the cancer surgery patients were older and more often male. Additionally, the preoperative heart rates for the two groups were comparable. Therefore, preoperative heart rates do not explain the difference in postoperative increased heart rate in cancer surgery. Interestingly, one might even expect lower heart rate values with increasing age [26, 27].

Limited clinical research on diurnal rhythm patterns in hospitalized patients is available. The diurnal rhythm in ICU patients was described in studies by Davidson et al. [13, 28]. Although planned postoperative patients were excluded, their figures show diurnal rhythm comparable to the findings in the present study. One of their main findings was the gradual increase in peak-nadir excursions during the ICU stay in the recovery group, which is consistent with the findings of the present study that show an increase for both surgery types. Additionally, diurnal rhythm patterns in vital parameters derived from continuous monitoring devices were studied in COVID-19 patients [29]. A diurnal rhythm was detected in the heart rate, respiration rate and temperature in recovering patients. The circadian rhythm influences various biological pathways, including heart rate, by coordinating rhythmicity in the suprachiasmatic nucleus (SCN) of the hypothalamus and peripheral clocks in tissues. The SCN synchronizes with the planetary cycle using external cues (zeitgebers) to regulate neurotransmitters and hormones [11]. Neurons in the SCN containing NMDA and GABA receptors affect clock gene expression. Many general anaesthesia drugs target these receptors, potentially affecting the molecular clock and rest/activity cycle and explaining the lack of diurnal rhythm in heartrate data in the first postoperative days [12]. The heart rate is known to be regulated through the autonomic nervous system as well via innervation of the sinus node through the release of neurotransmitters [30]. Significantly higher nadir and peak heart rates were observed in cancer surgery patients experiencing postoperative deterioration than in patients with deterioration-free recovery. In these comparisons, only data prior to the day of deterioration was included, therefor the composition of the population changes over time. Alterations in vital parameters are known to develop prior to deterioration. Therefore, diurnal rhythm features would also be suspected to be altered before deterioration [31]. The aforementioned study in ICU patients showed increased mean heart rate and lower peak nadir excursions in the nonsurvivors compared to survivors, concluding that patients who did not survive ICU admission had suppressed diurnal rhythm compared to those who did not [13]. In the first study describing diurnal rhythm in vital parameters in the general ward, rhythmicity was compared between a cohort of deteriorating COVID-19 patients and patients with normal recovery [29]. They observed decreased heart rate amplitude prior to deterioration, indicating a disturbed diurnal rhythm prior to deterioration. The findings of these studies match the observations in the present study, despite differences in the study populations.

Overall, resting heart rate appeared to be an important contributor to the results of the present study. Nadir heart rate in bariatric patients had a decreasing trend over the postoperative course, whereas peak heart rates remained at a more constant value. Consequently, increases in peak nadir excursions were mostly attributable to nadir heart rate. Resting heart rate might reflect the recovery status of the patient better, as it is less prone to alterations due to pain, stress or activity. Apart from diurnal rhythmicity, a decreasing resting heart rate could imply a trend towards recovery.

The present study is the first to describe the circadian rhythm using a wearable sensor to measure heart rate in postoperative patients, both during hospital stay and after discharge. The strengths of the present study are the observational design in which both researchers and the care team were blinded to the research data. This design reflected the real world patient population, including variation in the course of recovery. As the use of wearable sensors does not require action from nursing staff, apart from applying it once, the data collection was unobtrusive to both patients and clinical staff, allowing easy implementation for future use. Peak-nadir excursions were used to describe the diurnal rhythm, as they are intuitive and easy to implement in the processing of wearable sensor collected vital parameters.

Limitations of the present study include that no stratification for age or gender could be performed as the subgroups would have been too small. Second, the study population was heterogeneous in terms of comorbidity, preoperative condition and prehabilitation programmes, type of surgery, type of administered medication, length of stay, type of deterioration and postoperative recovery. In addition, the moment of diagnosis was set at the moment of documentation in the EMR, but treatments such as oxygen therapy or volume therapy could have been initiated before diagnosis was established. The impact of these pre-emptive treatments on vital parameters measured by the wearable device is unknown. Third, cohort-based results are presented. Future research should evaluate application at the individual patient level. Fourth, as the moment of deterioration was heterogeneous and could be well over a week after surgery, the difference between the deterioration and deterioration-free groups might be reduced, as larger differences in vital parameters are expected closer to the deterioration. Fourth, preoperative heart rate values were extracted from the EMR and while these values almost exclusively originated from daytime measurements during business hours, future studies could incorporate continuous preoperative monitoring to give more insight in the preoperative heart rate.

The results of the present study indicate the potential prognostic value of diurnal rhythm in postoperative heart rate to detect complications at an early stage. Novel wearable sensors allow for unobtrusive continuous monitoring of vital parameters, thereby allowing the monitoring of diurnal rhythm features as well. Recent publications imply that these sensors could provide an alternative for conventional EWS systems [14, 32]. Similar to conventional EWS systems, continuous EWS systems focus on the identification of abnormal vital parameters such as brady- or tachycardia. Implementation of diurnal rhythm in EWS systems could be achieved by adjusting the thresholds for heart rate. A recent paper by van Rossum et al. describes reducing the alarm threshold at night with the aim of avoiding false alarms [33]. Alternatively, incorporating diurnal rhythm by including an increased nadir heart rate, or a heart rate that is too high at night could be explored. However, prior to any application in clinical practice further research is required to study the viability of diurnal rhythm as a predictor of recovery or deterioration, the added value of adding diurnal rhythm in addition to the vital parameters that are currently incorporated in the early warning scores, as well as the feasibility of incorporating diurnal rhythm features in wearable-based early warning protocols.

## Conclusion

The postoperative diurnal rhythm of heart rate is disturbed by different types of surgery. All patients showed recovery of diurnal rhythm but in patients following major abdominal cancer surgery, both peak and nadir heart rates were higher than in patients following bariatric surgery. Especially nadir heart rate was identified as a potential prognostic marker for deterioration after cancer surgery.
